# Allergen immunotherapy of insect venom allergy: Almost 100 years old, but steadily updated 

**DOI:** 10.5414/ALX02420E

**Published:** 2023-12-12

**Authors:** Wolfgang Pfützner

**Affiliations:** Allergy Section, Department of Dermatology and Allergology, University Hospital Marburg, Philipps-Universität Marburg, Germany

**Keywords:** allergen immunotherapy, insect venom, allergy, IgE, sting provocation

## Abstract

llergen immunotherapy (AIT) with Hymenoptera venom (HV) shows high efficiency treating insect venom allergy, covering an almost 100-year-long history. Untreated patients with HV allergy can develop serious, potentially lethal sting reactions. Before starting AIT with HV, indication and contraindications, the presence of comorbidities and the intake of concomitant medications as well as individual risk factors have to be carefully evaluated. Application of HV-AIT entails an individually adapted procedure in case of undesired adverse events or initial failure to induce tolerance, as the final goal has to be the development of immunologic protection against anaphylactic sting reactions.

## Introduction 

Allergen immunotherapy (AIT) with insect venom looks back on an almost 100-year-old history. In September 1925, Dr. L.I.B. Braun reported on a woman who repeatedly had experienced severe anaphylaxis with unconsciousness after bee stings and who subsequently was successfully treated with an extract obtained from the terminal body section (~ 3 – 4 mm) of a bee [1]. This extract was first applied to the woman’s scarified skin and then injected in increasing doses, respectively. The process was subsequently modified in a way that whole body extracts were used [2]. Later on in 1956, in a study of patients with wasp allergy, Mary Hewitt Loveless was able to show that the development of tolerance was mediated by the contents of the venom sac [3]; however, it was another 20 years before AIT with the venom was confirmed as the only causally effective form of therapy for insect venom allergy, after another confirmation in a child [4], in a controlled study with a total of 41 adults [5]. Only 1 of 18 patients treated with wasp venom continued to show allergic symptoms after the sting challenge, in contrast to 7 of 12 treated with placebo and 7 of 11 receiving whole body extract. Equally convincing were the findings of a 1990 study with 242 children and adolescents aged 2 – 16 years. Here, only 1% of those stung in a follow-up period of 4 years after stopping AIT had another anaphylactic reaction, while this occurred in 18% of the untreated control group [6]. These results laid the basis for establishing AIT with wasp or bee venom as a very effective therapeutic method for inducing tolerance in IgE-mediated allergies to Hymenoptera venoms (HVs). This has been confirmed recently by a retrospective analysis of 1,258 patients with wasp or bee venom allergy, who were treated with 100 – 200 µg HV as a maintenance dose, with over 95% achieving tolerance to the sting challenge [7]. In addition to its high clinical effectiveness, HV AIT also leads to a significant improvement in quality of life [8]. 

## Indications and contraindications 

**Figure 1:**
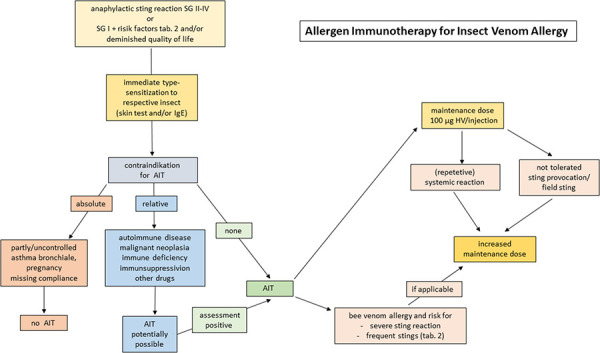
Procedure for the initiation and implementation of allergen immunotherapy with insect venom.

HV-AIT is indicated for patients with an anaphylactic sting reaction of severity grade (SG) > II (Table 1) or SG I when additional risk factors are present (Table 2) and/or the quality of life is impaired due to the allergy (Figure 1) [9]. The prerequisite is the detection of an IgE-mediated sensitization to the venom of the responsible insect by means of a positive skin test and/or detection of HV-specific IgE antibodies. In the case of double sensitization to bee and wasp venom, the component-based IgE analysis often enables a clear assignment [10, 11]. For children with an SG I sting reaction, data from various studies indicate that there may only be a low risk of renewed systemic reactions if no AIT is carried out [6, 12, 13]. For example, in a study of 2- to 16-year-olds with HV SG I anaphylaxis who did not receive AIT, 18% had another, also only mild, sting reaction [6]. In another study of children with a mean age of 8 (± 3) years, 13% of those not treated – compared to none of the children treated with AIT – with SG I anaphylaxis developed a sting reaction again over a follow-up period of up to 18 years; however, more than half of the second reactions showed an SG of II or III [12]. HV-AIT is not indicated for preventing excessive local reactions after a sting, defined as an erythematous swelling ≥ 10 cm in diameter that persists for several days (up to 3 weeks) and can be associated with systemic symptoms such as malaise and chills, especially in children [14, 15]. It is also not indicated for toxic or psycho-autonomic reactions, although the latter in particular often cannot be differentiated with certainty from anaphylactic symptoms [14]. 

Overall, there are only a few absolute contraindications for HV AIT. As stated in the general AIT guideline, it should not be carried out in the case of partially or uncontrolled bronchial asthma [16]. Likewise, it should not be initiated during pregnancy. However, if pregnancy occurs during maintenance therapy, HV-AIT can be continued in consultation with the expectant mother if it is well tolerated, particularly in view of the risk to the pregnancy in the event of another anaphylactic sting reaction [17]. Relative contraindications are the presence of autoimmune diseases, malignant neoplasms, immunodeficiencies, or the use of certain drugs. Stable and, in particular, organ-specific autoimmune diseases such as Hashimoto’s thyroiditis, inflammatory bowel disease, diabetes mellitus, or rheumatoid arthritis do not rule out HV-AIT, especially with regard to possible life-threatening sting reactions in allergic patients who have not been treated with AIT [9, 16, 17]. A tumor disease in remission does not necessarily have to be a contraindication to AIT [16, 17]. In this case, AIT should be coordinated with the responsible oncologists, taking into account the risk of relapse and metastasis on the one hand and the risk of stings and anaphylaxis on the other. Innate or acquired immune defects can limit the effectiveness of AIT, whose tolerance-inducing effect is based on immunomodulatory mechanisms such as the activation of regulatory T cells and the production of allergen-blocking antibodies [18]. In the case of HIV infection, however, if certain conditions are met (clinically stable disease under antiretroviral medication, normal CD4 count, negative HIV replication), HV-AIT can be effective and is indicated [16]. The same applies to carrying out HV-AIT under immunosuppressive medication, which is supported by data and experience with vaccinations [19]. It is assumed that long-term systemic administration of glucocorticosteroids with a prednisolone equivalent of < 20 mg/day, of methotrexate, or tumor necrosis factor-alpha inhibitors does not necessarily impair the development of a protective immune response [20, 21, 22]. 

The presence of cardiovascular disease and the use of beta blockers or ACE inhibitors are of particular importance when considering performing HV-AIT. For example, patients with HV allergy who also suffer from a cardiovascular (as well as chronic pulmonary) disease have an increased risk of severe sting reactions (Table 2). Achieving allergen tolerance and thus protection against sting anaphylaxis is therefore a high priority. Equally important is the optimal drug control of the underlying cardiac disease. However, there is debate as to whether beta blockers and ACE inhibitors have a negative effect on the course of an anaphylactic reaction to HV, the former through obstructive airway and circulatory depressive effects, the later through inhibition of kinin degradation [23]. Retrospective studies suggested that patients with HV allergy who received ACE inhibitors were more likely to suffer severe sting reactions [24, 25]. However, it cannot be ruled out that these reactions were primarily favored by the cardiac disease of those affected. Notably, several prospective studies did not detect any association between the intake of beta blockers and ACE inhibitors and the risk of severe anaphylactic reactions to the application of HV in the context of AIT [26, 27, 28]. It is therefore recommended for pragmatic reasons that, when performing HV-AIT, β-blockers can continue to be taken, but cardioselective preparations should be used, and ACE inhibitors should only be discontinued if switching to other preparations is possible without disadvantages for the treatment of the cardiac disease [9, 17]. 

If the product characteristics of the utilized AIT preparation contain information that deviates from the above-mentioned expert recommendations, this must be discussed with the person to be treated, explaining the individual advantages and disadvantages, and documented in the patients’ chart. 

## Procedure and therapy control 

For HV AIT, preparations with the venom of honey bees (*Apis melifera*) and wasps (*Vespula vulgaris* and *germanica*) are available throughout Europe. In southern countries, venom of the relevant paper wasps (*Polistes spp.*) can also be utilized. In the case of anaphylaxis after hornet or bumblebee stings, it is recommended to use the related wasp venom of the *Vespula* species and bee venom, respectively, if preparations of the reaction-triggering venom are not available [29]. Either native or purified aqueous compounds or aluminum- or tyrosine-adsorbed depot extracts can be employed for AIT, but these are not equally available in all European countries. In the build-up phase, the dosage is increased from an initially very small amount of HV of mostly 0.001 – 0.1 µg (whereby an initial dose of 1.0 µg in general is well tolerated [30]) to usually 100 µg HV/injection [9]. It should be considered to adjust patients with bee venom allergy to 200 µg/injection if there are risk factors for severe sting reactions or for more frequent sting events, e.g., if they are beekeepers (Table 3), since bees, in contrast to wasps, can release significantly more than 100 µg venom during a sting [31]. 

The up-dosing can be performed either exclusively with aqueous HV extracts within 1 – 2 or a few days by a (very) rapid dosage-increasing schedule (ultra-rush or rush AIT), or it can be performed via a cluster regimen or using the conventional outpatient procedure over several weeks [7, 32, 33]. The advantage of rapid up-dosing is the much faster achievement of clinical protection, which appears to be present in the majority of AIT-treated patients as early as 1 week after reaching the maintenance dose [34]. In the maintenance phase, injection intervals of 4 weeks are recommended for the first year of treatment; these can be extended to 6 weeks in the second year and to 8 weeks for depot preparations from the 3^rd^ year on [9]. 

As the most reliable method of therapy monitoring, a sting challenge can be performed during the course of AIT in centers that are appropriately equipped and experienced for this purpose [35, 36]. A tolerated sting challenge does not exclude with absolute certainty that a subsequent sting will again result in an allergic reaction. However, due to the controlled conditions that ensure an adequate sting by the allergy-causing insect, its validity is significantly higher than that of a sudden, unforeseen field sting [37]. In addition to confirming immunological protection, a tolerated sting is also associated with a noticeable improvement in the quality of life of the AIT-treated patient [38, 39]. Thus, an early provocation test should be aimed at. In addition, if a sting is not tolerated, measures can be taken early on to achieve HV tolerance. In a study of 79 bee venom-allergic patients who received a sting challenge 1 [eek after reaching the maintenance dose of 100 µg/injection, 89% exhibited tolerance, which underlines the rapid onset of protection [34]. In order to identify patients who may respond to HV AIT with a delay, and since hymenopterans are only available seasonally, still challenge is usually carried out ~ 6 – 12 [18] months after completion of the AIT build-up phase [35, 40]. The prerequisite is that the patient has tolerated the maintenance therapy without systemic reactions. Contraindications include pregnancy and comorbidities that are not adequately controlled by therapy, such as bronchial asthma or cardiovascular disease [35]. A sting challenge should not be carried out at the end of or even after completion of AIT, as there is a risk of boosting the allergen-specific IgE response resulting in reactivation of the HV allergy. If the challenge leads to an anaphylactic reaction, allergen tolerance can often be induced by increasing the HV dose to 1.5 – 2 times the previous maintenance dose [41]. 

In general, a duration of 5 years of HV AIT is recommended in order to ensure a long lasting therapeutic effect [9], which ideally has been demonstrated by a tolerated sting (sting challenge or, otherwise, field sting of the allergy-triggering insect). In case of an anaphylactic sting reaction during AIT with a subsequent dose increase, the treatment duration must be adjusted accordingly. Retrospective studies over a period of up to nearly 30 years after completion of HV AIT have shown that with increasing time interval from AIT, 10 – 20% of those treated lose the protection achieved, and this was particularly true for those who were re-stung more frequently after AIT [39, 42]. It is therefore recommended that patients with increased risk of expierencing Hymenoptera stings (e.g., outdoor occupation, beekeeping) continue HV AIT at least for the duration of the enhanced risk. If severe sting reactions are likely to occur in case of loss of tolerance (e.g., mastocytosis or index sting reaction SG IV), lifelong AIT should be carried out [9]. It is discussed whether in this case the injection intervals can be extended to 3 months, although more data on this subject, obtained in prospective studies are desirable. It should be borne in mind that in these cases – just as in the event of treatment with more than 100 µg HV (due to a dose increase or in the case of AIT with two allergen extracts) – higher (cumulative) doses of aluminum would be applied when aluminum-adsorbed depot preparations are used. Therefore, to be on the safe side, aqueous extracts should then be used instead [9]. 

## Tolerability and adverse events 

Observational studies show that even very rapid dose increases are generally well tolerated, both in adults and children [7, 32, 43, 44]. The possible adverse events can be divided into non-allergic reactions and allergic hypersensitivities. The former include local reactions at the injection site, which can be more pronounced when using non-purified preparations, and unspecific, common adverse events such as headaches and fatigue [45]. Allergic systemic reactions have been shown in multicenter studies in 8 – 20% of those treated [33, 46, 47]. They occur mainly in the induction phase, although this seems to be more common with rapid up-dosing, but there are no prospective comparative studies on this topic. Therapy with bee venom leads to systemic reactions significantly more frequently than treatment with wasp venom [30, 46, 48]. In contrast, in patients with mast cell diseases or elevated basal serum tryptase, HV AIT with wasp venom appears to be associated with a slightly higher risk of anaphylactic reactions [46]. Neither a recently published prospective study [26] nor retrospective studies [27, 28] found any indication for an increased risk of anaphylaxis in patients with cardiovascular disease or in those using β-blockers or ACE inhibitors. 

Most of the hypersensitive reactions to HV are not severe. In these cases, AIT can be continued at a dose reduced by two steps of the utilized AIT protocol and then increased again, trying to achieve a maintenance dose above the not tolerated dose [9]. AIT-accompanying, preventive administration of H1 antihistamines can be useful and prevent mild but not severe systemic reactions [26, 46]. In the case of repeated anaphylaxis following HV injection, predisposing factors, such as chronic infections, inadequately controlled bronchial asthma, or other potentially interfering diseases, must be ruled out. The temporary off-label use of the anti-IgE antibody omalizumab may also allow to successfully increase the dose or continue AIT [49]. It is also recommended to check whether the administration of a maintenance dose higher than 100 µg/injection is advisable in order to achieve sufficient protection against anaphylactic sting reactions (Table 3). In addition, it needs to be clarified whether AIT should be continued permanently, since various studies have shown that the risk of a loss of tolerance after the end of therapy is up to five times higher in patients experiencing anaphylactic sting reactions while receiving AIT [37, 50, 51]. 

## Conclusion 

AIT with HV poses special challenges for the allergist. This includes knowledge of the necessary prerequisites, risk factors to be considered, and adequate management of possible complications as well as patient-oriented communication and careful medical supervision of this therapy, which in certain cases can even be lifelong. If the special implications of HV AIT are taken into account, however, effective protection against insect sting-related anaphylaxis can almost always be achieved. 

## Conflict of interest 

The author has received honoraria from ALK-Abello (lectures) and Phadia/Thermo Fisher (lectures, research funding).

## Funding 

No funding. 

**Table 1.** Severity grades of anaphylaxis, modified according to Ring and Meßmer*.

SG	Skin	Gastrointestinal tract	Airways	Cardiovascular system
I	Flush, urticaria, angioedema	–	–	–
II		Nausea, cramps, urinary/fecal urgency	Rhinorrhea, hoarseness, difficulties swallowing, mild dyspnea	Vertigo, paleness, drop in blood pressure, mild circulatory symptoms
III		Vomiting, involuntary urination/defecation	Bronchospasm, severe dyspnea	Collapse/shock, unconsciousness
IV			Respiratory arrest	Cardiac arrest

SG = severity grade; no symptom is obligatory. *Ring J, Meßmer K. Incidence and severity of anaphylactoid reactions to colloid volume substitutes. Lancet. 1977; *1:* 466-469.

**Table 2.** Risk factors for repeated and severe sting reactions in Hymenoptera venom allergy.

Risk of more frequent stings	High occupational exposure to – Bees: e.g., beekeeping, horticulture – Wasps: e.g., bakery, forestry, road construction, fire brigade
Risk for more severe sting reactions	– Wasp stings – Mast cell diseases, mast cell tryptase > 11.4 µg/L – Instable bronchial asthma – Cardiovascular disease – Age > 40 years

**Table 3.** Indications for a maintenance dose of > 100 µg/injection.

– (Repeated) systemic reactions to maintenance dose
– Systemic reactions after sting challenge or field sting under AIT
– Possibly in case of bee venom allergy and risk of repeated bee stings or severe sting reactions (Table 2)
